# Imbalance of the spindle-assembly checkpoint promotes spindle poison-mediated cytotoxicity with distinct kinetics

**DOI:** 10.1038/s41419-019-1539-8

**Published:** 2019-04-05

**Authors:** Xiaofang Zeng, Wendy Kaichun Xu, Tsun Ming Lok, Hoi Tang Ma, Randy Y. C. Poon

**Affiliations:** 1 Division of Life Science, Center for Cancer Research, and State Key Laboratory of Molecular Neuroscience, Hong Kong University of Science and Technology, Clear Water Bay, Hong Kong; 2 Department of Oncology, The Third Xiangya Hospital, Central South University, Changsha, Hunan China; 3 Present Address: Department of Molecular Biosciences, Institute for Cellular and Molecular Biology, The University of Texas at Austin, Austin, TX USA

## Abstract

Disrupting microtubule dynamics with spindle poisons activates the spindle-assembly checkpoint (SAC) and induces mitotic cell death. However, mitotic exit can occur prematurely without proper chromosomal segregation or cytokinesis by a process termed mitotic slippage. It remains controversial whether mitotic slippage increases the cytotoxicity of spindle poisons or the converse. Altering the SAC induces either mitotic cell death or mitotic slippage. While knockout of MAD2-binding protein p31^comet^ strengthened the SAC and promoted mitotic cell death, knockout of TRIP13 had the opposite effect of triggering mitotic slippage. We demonstrated that mitotic slippage prevented mitotic cell death caused by spindle poisons, but reduced subsequent long-term survival. Weakening of the SAC also reduced cell survival in response to spindle perturbation insufficient for triggering mitotic slippage, of which mitotic exit was characterized by displaced chromosomes during metaphase. In either mitotic slippage or mitotic exit with missegregated chromosomes, cell death occurred only after one cell cycle following mitotic exit and increased progressively during subsequent cell cycles. Consistent with these results, transient inhibition of the SAC using an MPS1 inhibitor acted synergistically with spindle perturbation in inducing chromosome missegregation and cytotoxicity. The specific temporal patterns of cell death after mitotic exit with weakened SAC may reconcile the contradictory results from many previous studies.

## Introduction

Classic spindle poisons that either attenuate depolymerization (e.g. taxanes) or polymerization (e.g. vinca alkaloid) of microtubules are among the most useful chemotherapeutic agents available. Disrupting microtubule dynamics prevents proper attachment of microtubules to kinetochores, resulting in the activation of the spindle-assembly checkpoint (SAC) and mitotic arrest^1^.

Despite the widespread use of spindle poisons as front-line chemotherapeutic agents, precisely how they exert their cytotoxic effects remains perplexing. This is because the fate of cells after protracted mitotic block varies greatly between different cell lines as well as between individual cells from the same cell line^2^. The cell fate appears to be determined by two stochastically competing networks, one controlling mitotic cell death and the other mitotic slippage. On the one hand, mitotic cell death is believed to be caused by an accumulation of apoptotic activators and/or a loss of apoptotic inhibitors during mitosis^3^. On the other hand, it is possible for cells to exit mitosis into interphase without proper chromosome segregation and cytokinesis by a process termed mitotic slippage. The current paradigm states that an underlying mechanism of mitotic slippage is a slow degradation of cyclin B1 during mitotic arrest^4^.

Although mitotic slippage is a major outcome after antimitotic drug treatment, whether it promotes or reduces cytotoxicity remains a contentious issue. On the one hand, mitotic slippage interrupts the mitotic arrest and is expected to attenuate mitotic cell death. On the other hand, the tetraploid G_1_ cells generated after mitotic slippage are expected to be less fit to propagate than normal cells. The tetraploid DNA contents and supernumerary centrosomes generated after mitotic slippage can be further duplicated during the subsequent cell cycle and induce genome instability^5^.

An impressive number of studies in the literature contain experimental evidence either supporting that mitotic slippage increases the cytotoxicity of antimitotic drugs or the converse. On the one hand, many studies using diverse cell lines and methods of triggering mitotic slippage concluded that mitotic slippage limits the effectiveness of antimitotic drugs and promotes drug resistance. Examples include mitotic slippage induced by weakening of the SAC using small interfering RNAs (siRNAs) against MAD2 or BUBR1^6–8^, MAD2-targeting microRNA^9^, overexpression of p31^comet^ ^10, 11^ or MPS1 inhibitors^12^. Other approaches including expressing CDC6^13^, inhibiting aurora kinases^14–16^ or activating WEE1^17^ also reduced cytotoxicity of antimitotic drugs by inducing mitotic slippage. On the other hand, a number of studies indicate that mitotic slippage increases the effectiveness of antimitotic drugs. Examples include forcing mitotic slippage using CDK1 inhibitor^18–20^, aurora kinase inhibitor^21^, histone deacetylase inhibitor^22^, hyperthermia^23^, DNA damage^24^, siRNAs against survivin^25^ or BUBR1^26^, or inhibition of other targets^27^.

Why different studies on the effects of mitotic slippage, often using similar approaches, would give rise to such ambiguities and contradictions? If there are large gaps in our knowledge regarding the effects of mitotic slippage, even less is known about smaller scale of chromosomal instability such as missegregation of a small number of chromosomes. We suspect one possible explanation is the uncertainty of when cell fate should be measured after mitotic slippage. Given that mitotic slippage abolishes mitotic cell death, sampling shortly after mitotic slippage would result in an apparent increase in survival. The length of mitotic block could also affect post-exit cell death, presumably due to the accumulation of cell death signals during the arrest^15^. Furthermore, chemicals used to induce mitotic slippage may have non-specific effects and reduce viability during the subsequent interphase. Given these limitations, it is critical to understand precisely when cell death occurs after mitotic slippage, preferably inducing mitotic slippage without the use of chemicals. Here we used isogenic cell lines with different SAC status to study mitotic slippage more comprehensively. We monitored the events from immediately following mitotic slippage to over the next several days.

## Materials and methods

### Cell culture

The HeLa used in this study was a clone that expressed the tTA tetracycline transactivator^28^. HCT116 (colorectal carcinoma) was a gift from Bert Vogelstein (The Johns Hopkins University). TRIP13-knockout (TRIP13^KO^) cells, TRIP13^KO^-expressing FLAG-TRIP13 and p31^KO^ were generated as previously described^29^. TRIP13^KO^-expressing auxin-inducible degron (AID)-TRIP13 and TIR1 were generated as previously described^30^. Cells were propagated in Dulbecco’s modified Eagle’s medium supplemented with 10% (v/v) calf serum (for HeLa) or foetal bovine serum (for HCT116) and 50 U/ml of penicillin–streptomycin (Life Technologies, Carlsbad, CA, USA). Cells were cultured in humidified incubators at 37 °C with 5% CO_2_. Cell viability was assessed using trypan blue exclusion assay.

Unless stated otherwise, cells were treated with the following reagents at the indicated final concentration: doxycycline hydrochloride (Dox) (2 µg/ml), indole-3-acetic acid (IAA) (50 µg/ml), paclitaxel (PTX) (125 ng/ml), thymidine (2 mM) (Sigma-Aldrich, St. Louis, MO, USA), AZ3146 (0.5 µM), MG132 (10 µM) (Selleck Chemicals, Houston, TX, USA), nocodazole (NOC) (100 ng/ml), RO3306 (10 µM) and Z-VAD-FMK (20 µM) (Enzo Life Sciences, Farmingdale, NY, USA).

### Clonogenic survival assays

The indicated number of cells were plated onto 60-mm plates and allowed to grow overnight. The medium was then supplemented with vehicle alone or the indicated drugs for 24 h. Cells were washed gently with phosphate-buffered saline and fresh medium was replenished. After 2 weeks (unless stated otherwise, neither the drugs nor the medium was replenished over the period), colonies were fixed with methanol:acetic acid (v/v 2:1), followed by staining with 2% crystal violet. The number of colonies was quantified using Quantity One (Bio-Rad, Hercules, CA, USA).

### Cell cycle synchronization

Cell cycle synchronization using single or double thymidine method was performed as described^31^. Mitotic cells were synchronized using thymidine synchronization followed by NOC and isolated with mechanical shake-off as described previously^31^.

### Live-cell imaging and microscopy

Cells were seeded onto 24-well cell culture plates and placed onto a TE2000E-PFS microscope (Nikon, Tokyo, Japan) equipped with a SPOT BOOST EMCCD camera (SPOT Imaging Solutions, Sterling Heights, MI, USA) and an INU-NI-F1 temperature, humidity and CO_2_ control chamber (Tokai Hit, Fujinomiya, Shizuoka, Japan). Images were captured every 5 min (for 24 h imaging) or every 10 min (for >48 h imaging). Data acquisition was carried out with the Metamorph 7.5 software (Molecular Devices, Sunnyvale, CA, USA) and analysis was carried out manually using the ImageJ 1.52e software (National Institutes of Health). The fates of at least 50 cells (indicated in the Figures) were tracked through the entire imaging period for each condition. Following mitosis, one of the daughter cells (if there were more than one) was randomly chosen and tracked further.

### Flow cytometry analysis

Flow cytometry analysis after propidium iodide staining was performed as described previously^32^ except that a Coulter Epics XL flow cytometer was used (Beckman Coulter, Brea, CA, USA).

### Antibodies and immunological methods

Antibodies against β-actin (Sigma-Aldrich), phosphor-histone H3^Ser10^, TRIP13 (Santa Cruz Biotechnology, Santa Cruz, CA, USA) and cleaved PARP1(Asp214) (BD Transduction Laboratories, Franklin Lakes, NJ, USA) were obtained from the indicated suppliers. Antibodies against cyclin B1 were gifts from Julian Gannon (Cancer Research UK). Immunoblotting was performed as previously described^29^.

### Statistical analysis

Two-tailed Student’s *t* test was used to calculate statistical significance. *P* < 0.05 was considered statistically significant.

## RESULTS

### Altering the SAC induces cell death either during mitosis or after mitotic slippage

**Fig. 1:**
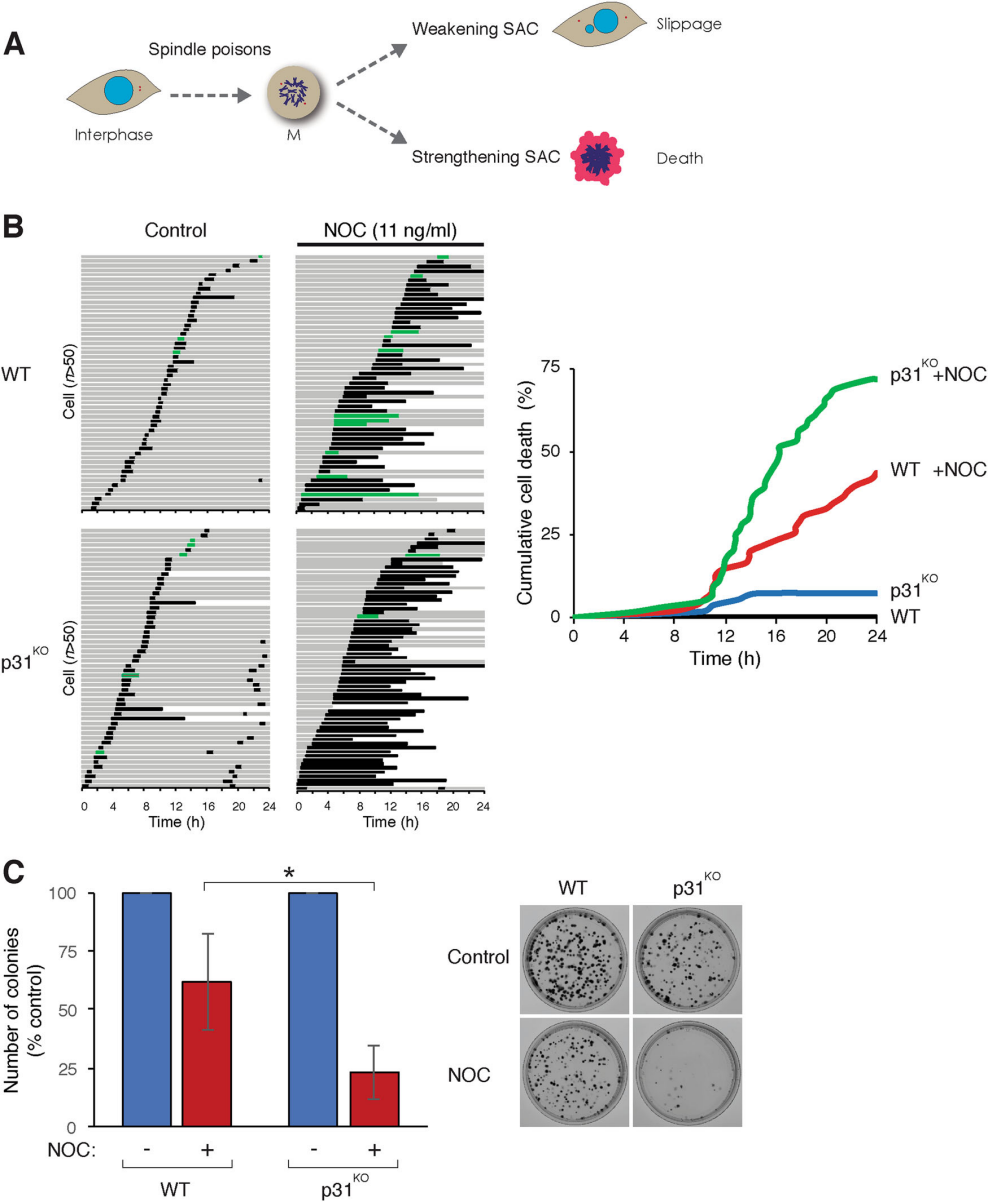
Delay in spindle-assembly checkpoint (SAC) inactivation sensitizes cells to spindle poisons. **a** Cell fate after spindle poison-induced mitotic arrest can be affected by the SAC. While weakening of the SAC promotes mitotic slippage, strengthening of the SAC promotes mitotic cell death. **b** Knockout of p31^comet^ promotes mitotic cell death. Wild-type (WT) and p31^comet^-deficient HeLa (p31^KO^)-expressing histone H2B-GFP were incubated with nocodazole (NOC) (11 ng/ml) and individual cells were tracked using live-cell imaging. Key: interphase = grey; mitosis (from DNA condensation to anaphase or cell death) = black; multipolar mitosis or mitosis with segregation errors = green; truncated bars = cell death. Cumulative cell death was quantified (right panel). **c** Knockout of p31^comet^ increases sensitivity to spindle poisons. WT and p31^KO^ were plated for clonogenic survival assay and incubated with NOC (11 ng/ml). The number of colonies was normalized to untreated control (mean ± SEM of three independent experiments). Representative images are shown on the right. *P < 0.05

The effectiveness of the SAC is believed to influence the outcome of spindle poison-mediated mitotic block (Fig. 1a). To strengthen the SAC without the use of chemical inhibitors, we used cells deficient in p31^comet^, which are impaired in turning off the SAC^29^. Wild type (WT) or p31^comet^-deficient HeLa cells expressing histone H2B-GFP were incubated with NOC and subjected to live-cell imaging (Fig. 1b). The relatively low concentration of NOC used in this experiment induced a delay without blocking mitosis in WT cells. By contrast, NOC induced a more prolonged mitosis in p31^comet^-deficient cells, ultimately resulting in mitotic cell death in the majority of the cells. Consistent with these results, clonogenic survival was reduced by NOC more significantly in p31^comet^-deficient cells than in WT cells (Fig. 1c). Similar results were obtained using WT and p31^comet^-deficient HCT116 cells, indicating that the effect was not limited to one cell line (Fig. S1).

**Fig. 2:**
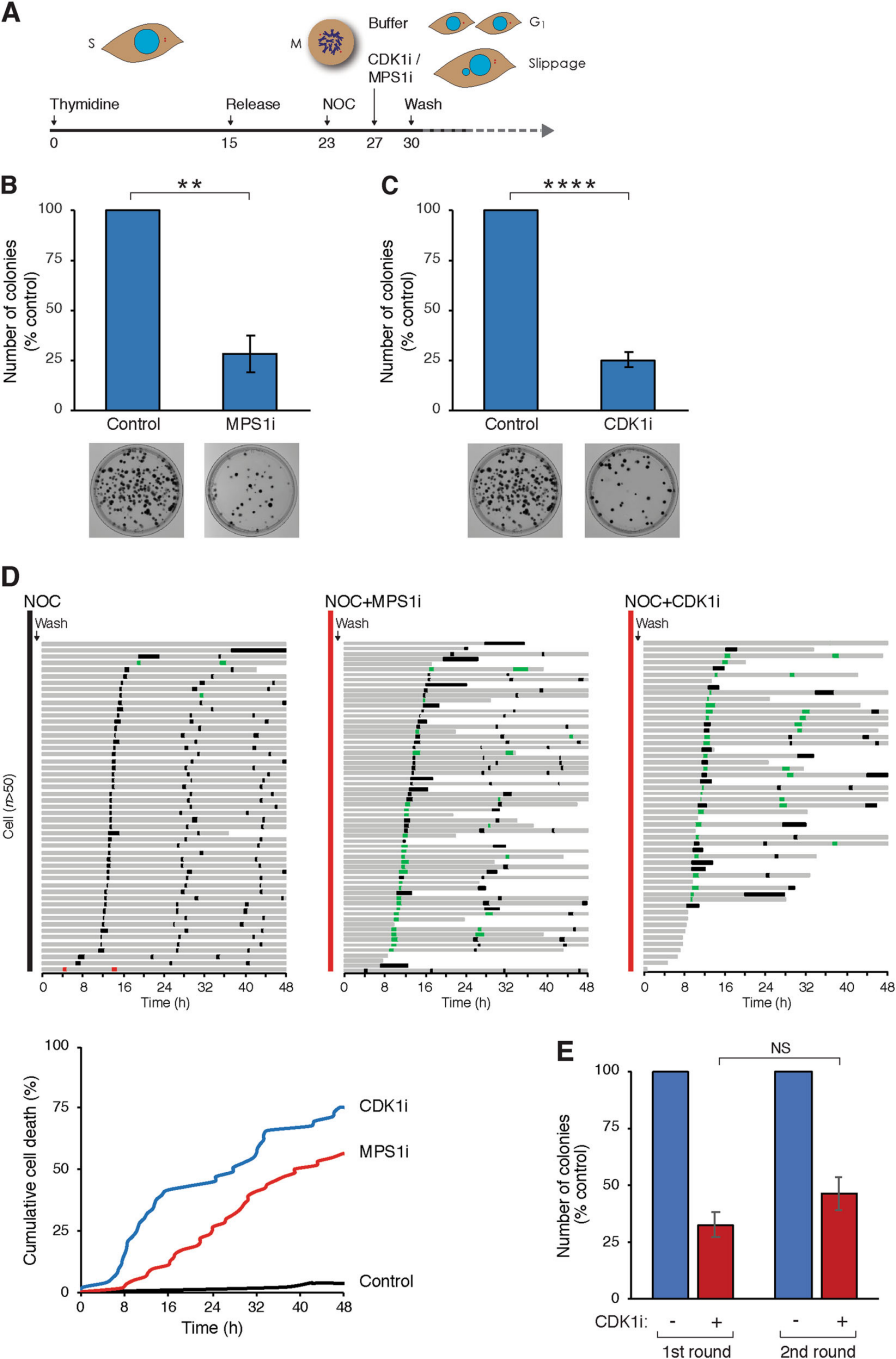
Chemical-induced mitotic slippage prevents immediate mitotic cell death but results in long-term cytotoxicity. **a** Schematic diagram of the synchronization procedure for inducing mitotic slippage. HeLa cells were synchronized at the S phase with thymidine for 15 h before releasing. At the G_2_ phase (8 h after release), the cells were incubated with nocodazole (NOC) (100 ng/ml). After 4 h, mitotic cells were collected by mechanical shake-off and treated with buffer (control), CDK1 inhibitor (CDK1i) or 1 µM of MPS1 inhibitor (MPS1i) for 3 h. After washing out the drugs and NOC, the cells were subjected to live-cell imaging or clonogenic survival analysis. **b** Mitotic slippage reduces cell survival. Cells were synchronized in mitosis and treated either with buffer or MPS1i to induce mitotic slippage (see **a**). After washing away NOC and MPS1i, equal number of cells were plated for clonogenic survival assay. The number of colonies was quantified (mean ± SEM of three independent experiments). Representative images are shown at the bottom. **c** Same experimental procedure as in **b**, except that CDK1i was used instead of MPS1i. **d** Cell death occurs progressively during subsequent cell cycles after mitotic slippage. HeLa cells expressing histone H2B-GFP were synchronized according to **a**. Mitotic cells were released from NOC block by washing and replating (control). Mitotic slippage was induced by incubating with MPS1i or CDK1i for 3 h (both NOC and MPS1i/CDK1i were then removed). Individual cells were then tracked using live-cell imaging for 48 h. Note that there was a short delay between the time of mitotic release or slippage and the beginning of imaging. Key: interphase = grey; mitosis = black; mitosis with mitotic slippage = red; multipolar mitosis or mitosis with segregation errors = green; truncated bars = cell death. Cumulative cell death was quantified (bottom panel). **e** Cells surviving mitotic slippage do not develop resistance to mitotic slippage. Cells were synchronized in mitosis and treated with CDK1i for clonogenic survival assay as described in **c**. After 3 weeks, the surviving cells were pooled and subjected to a second round of mitotic slippage induction. The number of colonies was quantified (mean ± SEM; *n* = 5 for first round and *n* = 3 for second round). NS not significant. **P < 0.01, ***P < 0.001

We next performed the converse experiments by prematurely inactivating the SAC. As the length of mitotic block could affect post-exit cell death^15^, synchronized culture was used to minimize the duration of NOC treatment (Fig. 2a). Note that a higher concentration of NOC was used compared to the previous experiment to induce a complete block in mitosis. We first uncoupled the SAC using an inhibitor of MPS1 (MPS1i), which induced cyclin B1 degradation (thereby inactivating CDK1) and mitotic exit as indicated by dephosphorylation of histone H3^Ser10^ (Fig S2). Alternatively, CDK1 was directly inactivated using the CDK1 inhibitor RO3306 (CDK1i). In both cases, mitotic exit occurred rapidly without chromosomal segregation or cytokinesis. Importantly, clonogenic survival was significantly reduced after mitotic slippage in comparison to cells released from the mitotic block (Fig. 2b, c).

The time of cell death after mitotic slippage was visualized directly using live-cell imaging (Fig. 2d). Within the 48 h imaging period, nearly all WT cells were able to complete three cell cycles after they were released from NOC-mediated arrest. By contrast, although the majority of cells remained viable during the cell cycle immediately post-mitotic slippage, cell death increased steadily after the subsequent mitosis (Fig. 2d, bottom panel).

As a prevailing view is that mitotic slippage increases resistance to spindle poisons (see Introduction), we further explored if cells surviving mitotic slippage have different sensitivity to spindle poisons or further rounds of mitotic slippage. Mitotic slippage was induced by treating cells with NOC followed by CDK1i. The surviving cells were then pooled and expanded. These cells displayed similar sensitivity to NOC as the WT cells (data not shown). Moreover, they were killed by a second round of mitotic slippage to a similar extent as WT cells (Fig. 2e), indicating that cells surviving mitotic slippage did not develop further resistance to spindle poisons.

**Fig. 3:**
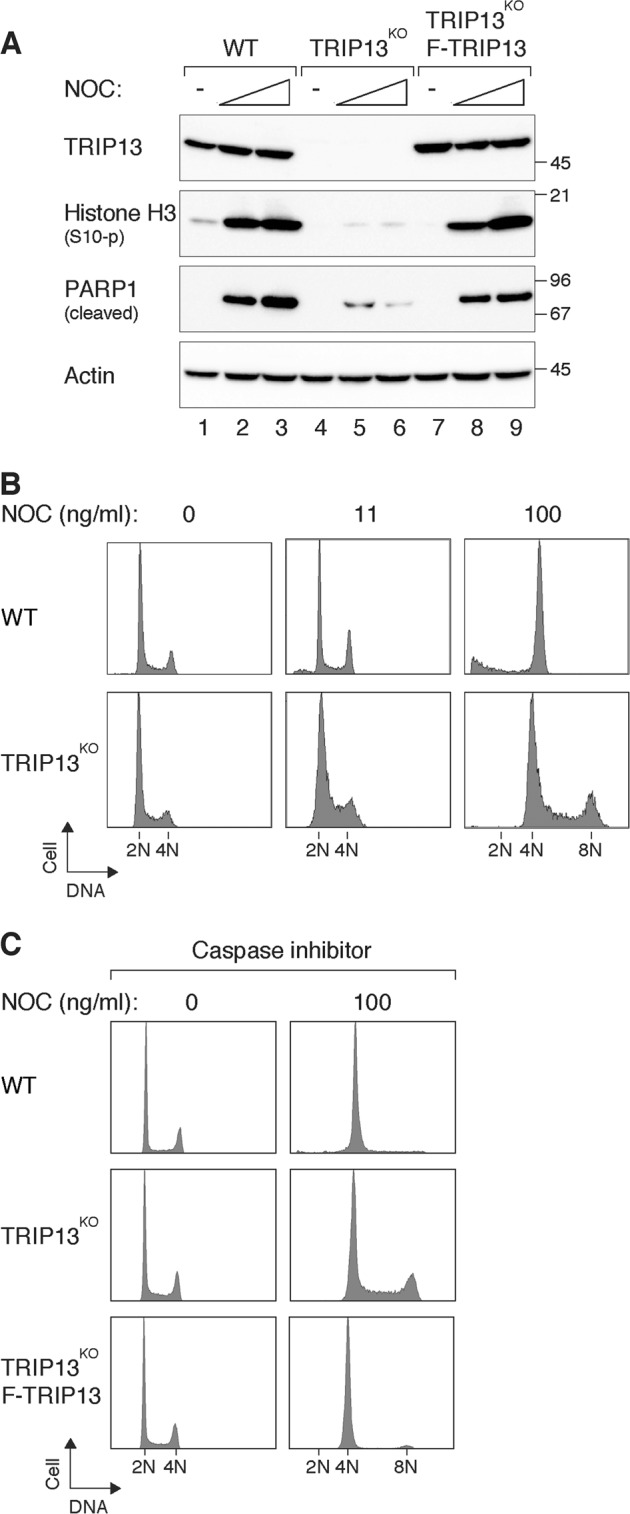
Spindle-assembly checkpoint (SAC)-deficient cells resist mitotic cell death through mitotic slippage. **a** TRIP13 deficiency promotes mitotic slippage and reduces mitotic cell death. HeLa (wild-type (WT)), TRIP13-knockout (TRIP13^KO^) cells, and TRIP13^KO^-expressing FLAG-TRIP13 were treated with buffer or nocodazole (NOC) (33.3 and 100 ng/ml) for 24 h. Lysates were prepared and the expression of the indicated proteins was analysed with immunoblotting. Uniform loading of lysates was confirmed by actin analysis. Molecular size markers (in kDa) are shown on the right. **b** TRIP13 deficiency promotes re-replication. WT and TRIP13^KO^ cells were exposed to buffer or the indicated concentrations of NOC for 24 h. The cells were then fixed and analysed with flow cytometry. The positions of 2*N*, 4*N* and 8*N* DNA contents are indicated. **c** WT, TRIP13^KO^ cells and TRIP13^KO^-expressing FLAG-TRIP13 were incubated with buffer or NOC in the presence of a pan-caspase inhibitor. After 24 h, the cells were fixed and analysed with flow cytometry

A caveat of the above experiments is that we cannot exclude the possibility that cell death occurred after CDK1i or MPS1i treatment was caused by the toxicity of the chemicals in addition to mitotic slippage. Consistent with that, CDK1i and MPS1i promoted cell death at different rates post-mitotic slippage (Fig. 2d). To circumvent this, we made use of cells engineered to have a defective SAC. Impaired SAC, however, undermines viability even in the unperturbed cell cycle. For example, depletion of MAD2 results in inviability in HeLa^30^ or mouse models^33^. TRIP13 is involved in converting MAD2 from the active C-MAD2 to the inactive O-MAD2 conformation^29^. Although the newly synthesized O-MAD2 in TRIP13-deficient cells is sufficient to support unperturbed cell cycle, the predominantly C-MAD2 environment is insufficient to enable the SAC after challenged with NOC^30^. Individuals with loss-of-function mutations in TRIP13 show dysregulated SAC and are predisposed to Wilms’ tumour^34^. In contrast to HeLa cells, which could be arrested in mitosis with NOC, TRIP13^KO^ cells failed to be trapped in mitosis (Fig. 3a). Importantly, apoptosis occurred in WT cells, but not in TRIP13^KO^ cells (as indicated by cleavage of PARP1). Both mitotic block and cell death could be rescued by re-introducing FLAG-TRIP13 into the TRIP13^KO^ cells.

The evasion of mitotic cell death in TRIP13^KO^ cells was further confirmed using flow cytometry analysis (Fig. 3b). While WT cells were arrested in G_2_/M after being challenged with NOC for 24 h, TRIP13^KO^ cells also contained 8*N* DNA contents, indicating they underwent mitotic slippage followed by DNA replication. As a portion of NOC-treated WT cells underwent apoptosis (Fig. 3a, b), a pan-caspase inhibitor was included to confirm that WT cells did not undergo mitotic slippage and DNA replication in the absence of apoptosis (Fig. 3c).

Mitotic cell death induced with another spindle poison, PTX, was also abolished in TRIP13^KO^ cells, indicating that the effects were not limited to NOC (Fig. S3). We further performed similar analyses using TRIP13^KO^ cells generated from HCT116 (Fig. S4a), confirming that the abolition of mitotic cell death by TRIP13 deficiency was not cell line-specific (Fig. S5).

**Fig. 4:**
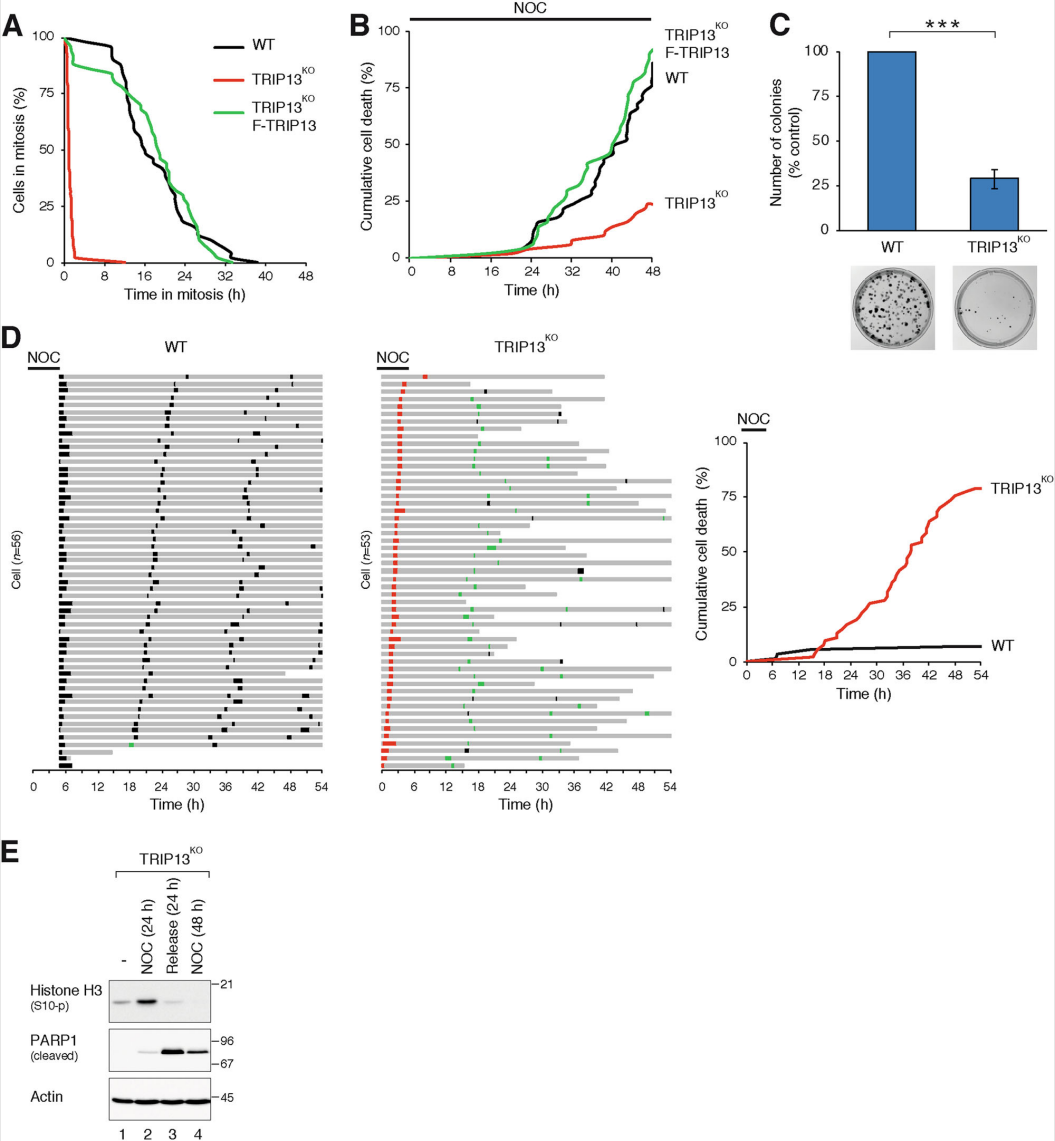
Cell death starts to occur after one cell cycle following mitotic slippage. **a** TRIP13-deficient cells undergo rapid mitotic exit. HeLa (wild-type (WT)), TRIP13-knockout (TRIP13^KO^) cells and TRIP13^KO^-expressing FLAG-TRIP13 (all expressing histone H2B-GFP) were exposed to nocodazole (NOC) and analysed using live-cell imaging. The duration of mitosis in individual cells was quantified (from DNA condensation to mitotic slippage or cell death). The live-cell imaging profile of individual cells in the analysis is shown in Fig. S6a. **b** Mitotic slippage delays NOC-mediated cell death. Cells were exposed to NOC and analysed with live-cell imaging as described in **a** to quantify cumulative cell death. **c** Mitotic slippage reduces long-term survival. WT and TRIP13^KO^ cells were synchronized using a double thymidine block procedure and treated with NOC for 6 h. After washing, the cells were plated for clonogenic survival assay. The number of colonies was normalized to control. Mean ± SEM of three independent experiments. Representative images are shown at the bottom. **d** Mitotic slippage results in cell death after the subsequent mitosis. WT and TRIP13^KO^-expressing histone H2B-GFP were synchronized using a double thymidine block procedure and treated with NOC. After 1 h of NOC treatment, the cells were subjected to live-cell imaging analysis. NOC was washed out after another 5 h before continue imaging. Because WT cells did not attach well to the plate in the presence of NOC, they were only imaged after NOC was removed. Key: interphase = grey; mitosis = black; mitosis with mitotic slippage = red; multipolar mitosis or mitosis with segregation errors = green; truncated bars = cell death. Cumulative cell death was quantified (right). **e** Releasing cells into normal media after mitotic slippage triggered more cell death than continue incubation in NOC. TRIP13^KO^ cells were incubated with NOC for 24 h. One portion of the cells was harvested. Another portion was either incubated with NOC or released into NOC-free medium before harvesting after another 24 h. The cells were analysed with immunoblotting for cleaved PARP1. ***P < 0.001

Finally, directly observing mitotic slippage using live-cell imaging revealed that while WT cells were able to be trapped in mitosis by NOC (mean ~16 h), TRIP13^KO^ cells rapidly underwent mitotic slippage (Fig. 4a). An example is shown in Supplemental video 1. The mitotic block was restored in TRIP13^KO^ cells with recombinant TRIP13. Similar results were obtained using PTX as a mitotic blocker (Fig. S6b). Collectively, these data demonstrated that mitotic slippage prevents mitotic cell death caused by spindle poisons, but reduces subsequent long-term survival.

### Cell death occurs only after one cell cycle following mitotic slippage

The above data indicate that SAC deficiency promoted short-term survival (up to a few cell cycles) following treatment with NOC or PTX (Fig. 4b, Fig. S6c). Paradoxically, long-term viability (up to a few weeks) was reduced in SAC-deficient cells (Fig. 4c). To determine more precisely the timing of cell death, synchronized cells were treated with NOC followed by live-cell imaging spanning several cell cycles (Fig. 4d). As before, while WT cells could be arrested in mitosis with NOC, TRIP13^KO^ cells underwent rapid mitotic slippage. After washing out the NOC, WT cells underwent about three rounds of cell cycle without exhibiting significant level of cell death. Interestingly, tetraploid TRIP13^KO^ cells remained viable during the first cell cycle post-mitotic slippage. Cell death only started to accumulate after the first mitosis following mitotic slippage. As anticipated, the majority of the mitoses after mitotic slippage were characterized by abnormalities, including multipolar mitosis and chromosome missegregation (>80%). Cell death, however, occurred predominantly during interphase and at an approximately constant rate.

The above data suggested that mitotic slippage-mediated whole-genome imbalance was not the main cause of cell death. Indeed, less cell death occurred in TRIP13^KO^ cells if NOC was applied continuously (mitotic slippage also occurred in subsequent mitoses) compared to when NOC was removed after mitotic slippage as detected by both live-cell imaging (compare Fig. 4d and Fig. S6a, respectively) and the appearance of cleaved PARP1 (Fig. 4e).

Collectively, these results indicate that while mitotic slippage prevents cell death through the first cell cycle, cell death occurs progressively during subsequent cell cycles.

### Defective SAC also reduces cell survival in response to spindle perturbation insufficient for mitotic slippage

**Fig. 5:**
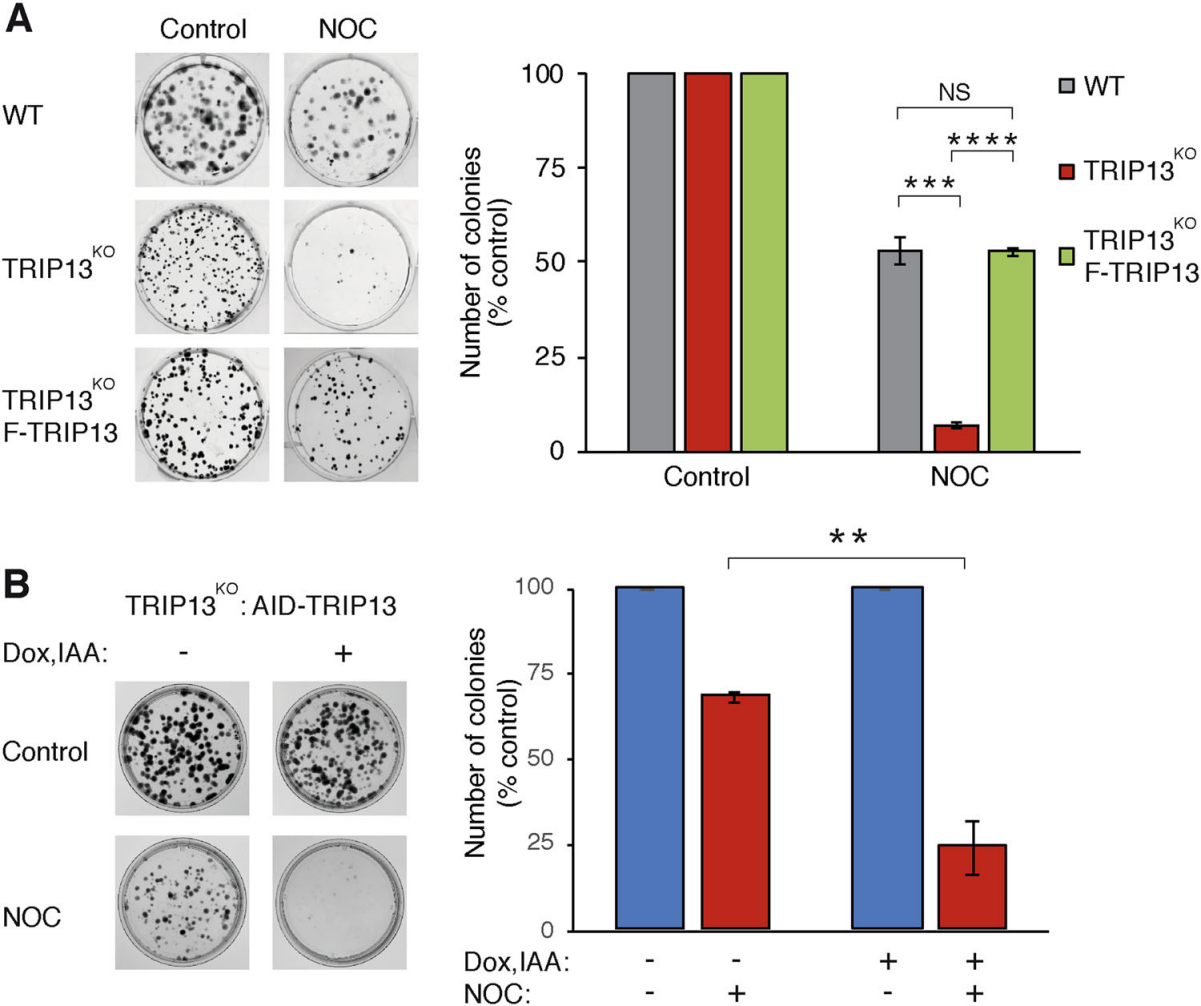
Spindle-assembly checkpoint (SAC)-deficient cells are sensitive to partial spindle perturbation. **a** SAC-deficient cells are sensitive to a sub-lethal dose of nocodazole (NOC). Equal number of HeLa (wild-type (WT)), TRIP13-knockout (TRIP13^KO^) cells and TRIP13^KO^-expressing FLAG-TRIP13 were treated with either buffer or NOC (11 ng/ml) for 24 h. After gentle washing, the cells were grown for another 14 days. Colonies were fixed, stained (representative images are shown on the left), and quantified (right). Mean ± SEM of three independent experiments. **b** Inducible depletion of TRIP13 promotes sensitivity to NOC. TRIP13^KO^ cells expressing auxin-inducible degron (AID)-TRIP13 were grown in the presence or absence of indole-3-acetic acid (IAA) and doxycycline hydrochloride (Dox) for 36 h. The cells were treated with NOC (11 ng/ml) for 24 h before gently washing. IAA and Dox were replenished in the group with the treatment. Colonies were fixed and stained after 14 days. The number of colonies was quantified (mean ± SEM of three independent experiments). Representative images are shown on the left. NS not significant. ***P < 0.001, ****P < 0.0001

Given that the effective concentration of spindle poisons achieved in vivo is unlikely to completely disrupt spindles or induce a prolonged mitotic arrest (reviewed in ref. ^3^), we next studied the temporal dependency of cytotoxicity after SAC-deficient cells were treated with low concentrations of spindle poisons. We found that 11 ng/ml of NOC induced neither a significant change in cell cycle profile (Fig. 3b) nor any mitotic slippage as observed using live-cell imaging (see later) in SAC-proficient or -deficient cells. Nonetheless, incubation of TRIP13^KO^ cells with this concentration of NOC reduced clonogenic survival by >90% (compared to ~50% in WT cells) (Fig. 5a). TRIP13-deficient HCT116 cells were also more sensitive to NOC than WT cells (Fig. S7a). Sensitivity to a sublethal dose of PTX was likewise increased by TRIP13 deficiency (Fig. S7b).

Although WT and TRIP13^KO^ cells were isogenic, it is conceivable that additional genetic or epigenetic changes may have occurred during the generation of these cell lines. To ensure that the effects of spindle poisons were due to TRIP13 deficiency alone, we also used TRIP13^KO^ cells expressing AID-tagged TRIP13 to conditionally inactivate TRIP13. AID-tagged proteins are degraded rapidly in response to plant auxin (or IAA) in cells expressing the ubiquitin ligase SCF^TIR1 ^^35^. Consequently AID-TRIP13 could be shut off in the presence of IAA and doxycycline (the AID-TRIP13 was also under the control of a Tet-Off promoter) (Fig S4b). Figure 5b shows that less number of colonies were formed after AID-TRIP13 degradation, confirming that SAC-deficient cells were more sensitive to low concentrations of NOC.

**Fig. 6:**
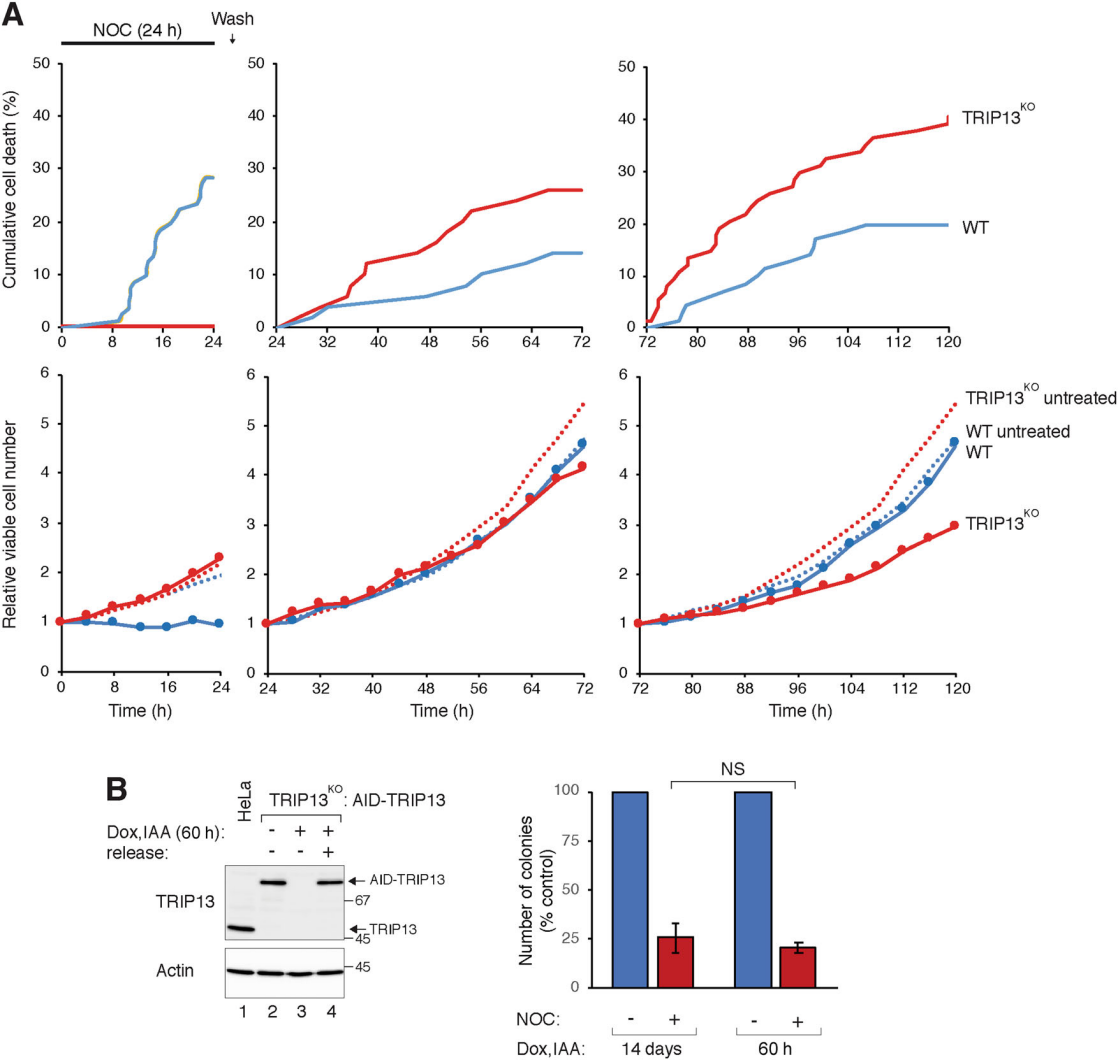
Aberrant mitosis induced by defective spindle-assembly checkpoint (SAC) and partial spindle perturbation promotes cell death after the following cell cycle. **a** HeLa (wild-type (WT)) and TRIP13-knockout (TRIP13^KO^)-expressing histone H2B-GFP were exposed to nocodazole (NOC) (11 ng/ml) for 24 h and analysed with live-cell imaging. The cells were washed and subjected to another period of live-cell imaging (24–72 h after initial NOC addition). Another batch of cells treated with NOC followed by washing was imaged from 72 to 120 h after initial NOC addition. Cumulative cell death over the individual imaging period was quantified (upper panels). The live-cell imaging profile of individual cells in the analysis is shown in Fig. S8. The changes in total number of viable cells after NOC treatment was also estimated from the live-cell imaging data (solid lines) and compared to that of untreated control (dotted lines). **b** The lack of SAC after the initial stage of spindle perturbation is not critical for subsequent cytotoxicity. TRIP13^KO^ cells expressing auxin-inducible degron (AID)-TRIP13 were grown in the presence of indole-3-acetic acid (IAA) and doxycycline hydrochloride (Dox) for 36 h. The cells were treated with either buffer or NOC (11 ng/ml) for 24 h before gently washing. IAA and Dox was then added back to one of the plates. A portion of the cells were plated for clonogenic survival assay (mean ± SEM of three independent experiments). The rest of the cells were harvested for preparing lysates and immunoblotting analysis (left). Lysates from the parental HeLa cells were loaded to indicate the position of the endogenous TRIP13. NS not significant

To determine when cell death was initiated, live-cell imaging was used to analyse cells both during NOC exposure and at different periods after washing (Fig. 6a). During incubation with NOC (24 h), cell death occurred exclusively in WT cells, but not TRIP13^KO^ cells. The majority of cell death occurred during mitosis (>90%), which was substantially longer than unperturbed mitosis (Fig. S8). In marked contrast, TRIP13^KO^ cells displayed negligible level of cell death in the presence of NOC as well as during the first cell cycle after release (Fig. S8). Significantly, more cell death occurred in TRIP13^KO^ cells than in WT cells over the subsequent cell cycles (of which >90% occurred during interphase) (Fig. 6a). The switch in the level of cytotoxicity between SAC-proficient and -deficient cells during and after NOC treatment was also reflected in the total cell number. In the presence of NOC, there was an increase in cell number in TRIP13^KO^ cells, but not in WT cells. WT and TRIP13^KO^ cells then proliferated at the same rate over the next 48 h. Finally, WT cells proliferated more rapidly than TRIP13^KO^ cells over the following 48 h, reflecting the increase in cytotoxicity in TRIP13^KO^ cells at later cell cycles (Fig. 6a).

An interesting question is whether the sensitization to spindle perturbation was due to defective SAC during the initial period of NOC challenge or also over the subsequent cell cycles. To demarcate these possibilities, AID-TRIP13 was turned off either for only the period of NOC treatment (24 h) or for the entire period of clonogenic formation (14 days). Figure 6b shows that AID-TRIP13 was able to re-accumulate after Dox and IAA were removed. Moreover, a similar number of colonies were formed irrespective of whether TRIP13 was present or absent during the subsequent recovery period, suggesting that the lack of SAC during spindle perturbation is more critical for the cytotoxicity than the subsequent cell cycles.

**Fig. 7:**
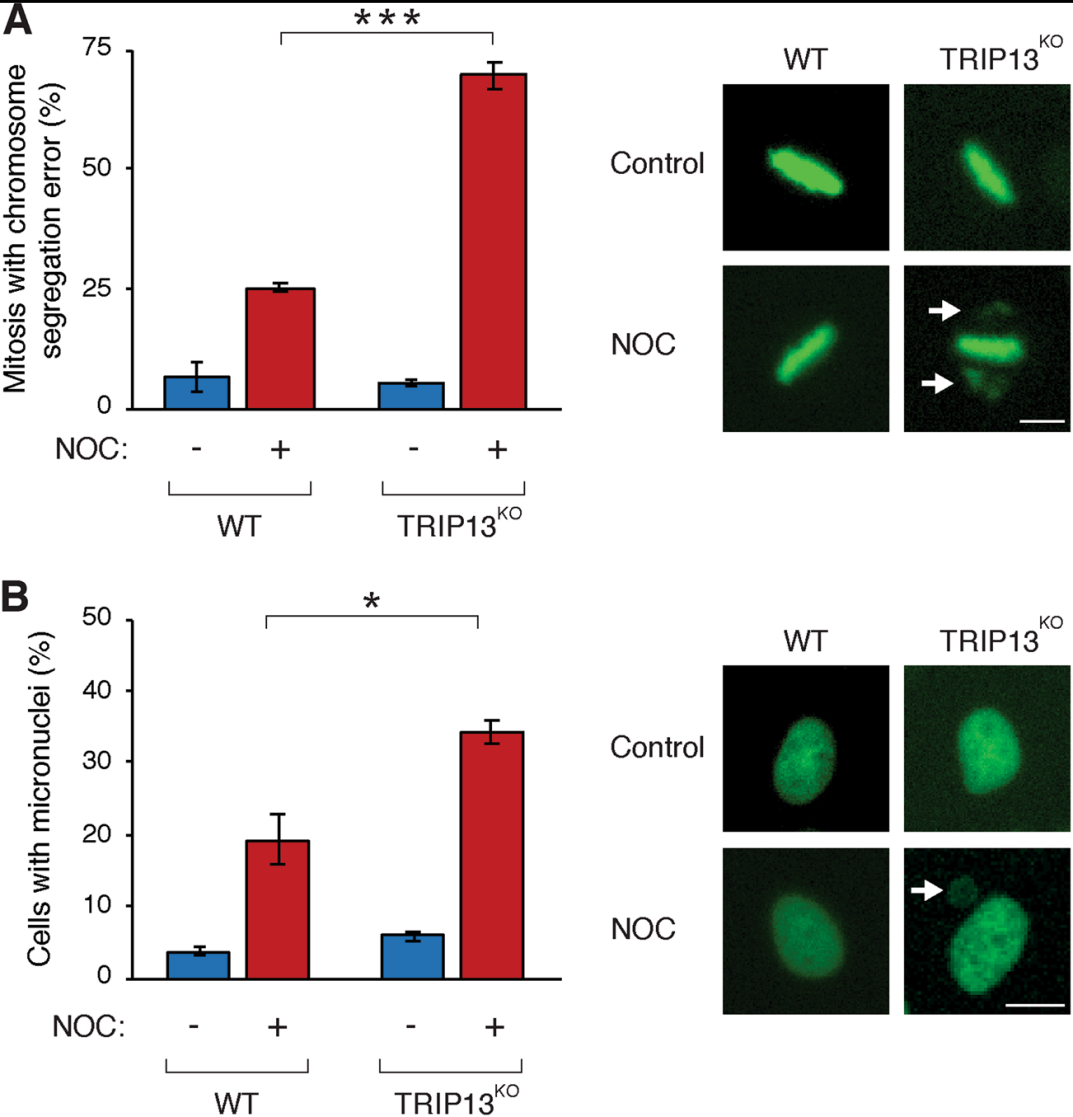
Mitosis induced by defective spindle-assembly checkpoint (SAC) and partial spindle perturbation is characterized by chromosome missegregation. **a** TRIP13 deficiency increases displaced chromosomes during metaphase upon partial spindle perturbation. HeLa (wild-type (WT)) and TRIP13-knockout (TRIP13^KO^)-expressing histone H2B-GFP were exposed to either buffer or nocodazole (NOC) (11 ng/ml) and analysed with live-cell imaging. The percentage of mitoses with displaced chromosomes during metaphase was quantified (mean ± SEM of three independent experiments). Representative images of metaphase are shown on the right (arrows indicate displaced chromosomes; the cell went on to undergo anaphase without aligning these chromosomes). Scale bar: 10 µm. **b** Mitosis in TRIP13-deficient cells upon partial spindle perturbation results in an increase in micronuclei. Experiments were performed as described in **a**. The percentage of cells containing micronuclei after mitosis was quantified (mean ± SEM of three independent experiments). Representative images are shown on the right (arrow indicates a micronucleus). Scale bar: 10 µm. *P < 0.05, ***P < 0.001

Although partial spindle perturbation did not induce mitotic slippage in TRIP13^KO^ cells, mitosis was characterized by chromosome missegregation. More than 60% of mitoses contained displaced chromosomes during metaphase (Fig. 7a, Fig. S8), which frequently resulted in micronucleation in the daughter cells (Fig. 7b). An example is shown in Supplemental video 2.

Collectively, these results indicate that weakening of the SAC promotes either mitotic slippage or chromosome missegregation, depending on the concentration of spindle poison used. Both result in cell survival over the first cell cycle, but progressively increase in cell death during subsequent cell cycles.

### Chemical inhibition of SAC promotes chromosomal missegregation and reduced viability

**Fig. 8:**
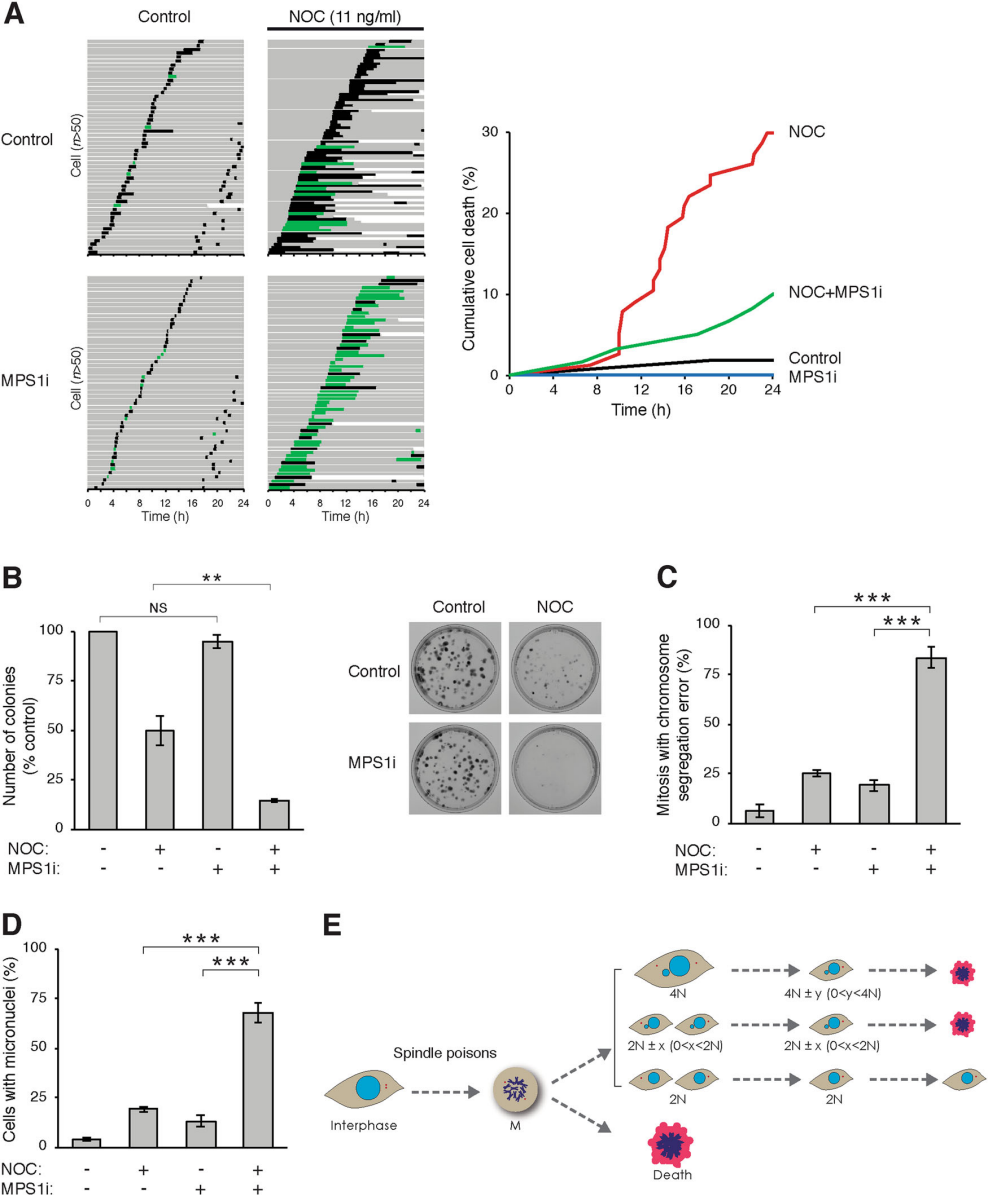
Overriding the spindle-assembly checkpoint (SAC) with MPS1 inhibitor transiently increases survival and reduces long-term viability. **a** MPS1 inhibitor (MPS1i) prevents nocodazole (NOC)-mediated mitotic cell death. HeLa cells expressing histone H2B-GFP were incubated with 0.5 µM of AZ3146 (MPS1i) and/or 11 ng/ml of NOC before analysing with live-cell imaging. Key: interphase = grey; normal mitosis = black; multipolar mitosis and mitosis with segregation errors = green; truncated bars = cell death. Cumulative cell death was quantified (right). **b** MPS1i in the presence of spindle perturbation reduces long-term survival. HeLa cells were treated with the indicated combination of MPS1i and/or NOC for 24 h before gently washing. Colonies were fixed and stained after 14 days. The number of colonies was quantified (mean ± SEM of three independent experiments). Representative images are shown on the right. **c** Inhibition of MPS1 enhances chromosome segregation error. Cells treated with MPS1i and/or NOC were analysed with live-cell imaging as described in **a**. The percentage of mitosis with chromosome segregation errors was quantified (>50 mitotic cells were analysed in each experiment; mean ± SEM of three independent experiments). **d** Inhibition of MPS1 increases daughter cells containing micronuclei. Cells treated with MPS1i and/or NOC were analysed with live-cell imaging as described in **a**. After mitosis, the percentage of daughter cells containing micronuclei was quantified (>50 mitotic cells were analysed in each experiment; mean ± SEM of three independent experiments). **e** Cell fate following spindle and SAC disruption. Cells that survive spindle poison-mediated mitotic delay can undergo mitotic slippage (forming tetraploid cells with 4*N* DNA contents), unequal cell division (caused by chromosome segregation error) or normal cell division into two daughter cells. Due to the supernumerary centrosomes, unequal cell division can also occur during subsequent cell cycles after mitotic slippage. In cancer cell lines such as HeLa, the gain or loss in chromosome number after aberrant mitosis (*x* and *y* in the figure) does not result in immediate cell death. Instead, loss of viability occurs progressive over the subsequent cell cycles. NS not significant. **P < 0.01, ***P < 0.001

The above results showed that the SAC requires to be inactivated only transiently during spindle perturbation to promote subsequent cell death (Fig. 6b). Given its clinical implication, we further characterized if transient inhibition of the SAC with small chemical inhibitors could recapitulate the chromosome missegregation and the temporal pattern of cytotoxicity as SAC-deficient cells. Inhibition of MPS1 with RNA interference or inhibitors increases the sensitivity to spindle poisons^36^. Using live-cell imaging analysis, we found that MPS1i reduced the short-term cell death caused by NOC (Fig. 8a). Note that MPS1i did not trigger mitotic slippage in the presence of the relatively low concentration of NOC. However, MPS1i and NOC together reduced long-term clonogenic survival more significantly than the individual drug alone (Fig. 8b). MPS1i treatment correlated with an increase in displaced chromosomes during metaphase (Fig. 8c) and micronuclei in daughter cells (Fig. 8d). An example in shown in Supplemental video 3. Collectively, these results indicate that chemical inhibition of the SAC acts synergistically with spindle perturbation in inducing chromosome missegregation and cytotoxicity.

## DISCUSSION

In this study, we evaluated the temporal regulation of cell death following aberrant mitosis, intending to reconcile the number of conflicting reports concerning the effect of SAC on the effectiveness of spindle poisons. SAC-hyperactive (p31^KO^) or SAC-deficient (TRIP13^KO^) cells were used in the study, thereby avoiding possible non-specific effects associated with chemical inhibitors. In SAC-hyperactive p31^KO^ cells, mitotic cell death became the predominant cell fate following exposure to even low concentrations of spindle poisons (Fig. 1). By contrast, SAC-deficient TRIP13^KO^ cells prevented mitotic cell death by accelerating mitotic slippage or mitotic exit with chromosome missegregation.

Within the first 48 h treatment with spindle poisons, cell death was reduced in cells undergoing mitotic slippage compared to those arrested continuously in mitosis (Fig. 4b). During the subsequent several cell cycles, the continued presence of spindle poisons resulted in mitotic slippage in the subsequent mitoses, reducing the overall cell death (Fig. 4e, Fig. S6a). But if spindle poisons were removed after mitotic slippage, cell death began to accumulate after the following mitosis, occurring mainly during interphase (Fig. 4d, e). As the spindle poisons were already removed, the cell death was independent on the direct toxicity of the spindle poisons during interphase^37^. This was consistent with the diminishing long-term cell survival after mitotic slippage (Fig. 4c). The progressive reduction in viability was likely to be due to chromosomal instability caused by the subsequent uncoordinated centrosome cycle and multipolar mitosis (Fig. 4d). In addition, DNA damage can accumulate during the aberrant mitosis^38, 39^ and may be responsible for some of the apoptosis during the subsequent interphase^40^. The time involved in switching from abrogating cell death immediately post-mitotic slippage to increasing cell death over the subsequent cell cycles may reconcile the contradictory results obtained in different studies in the literature (see Fig. 8e for model).

In spite of having an extra set of chromosomes, post-mitotic slippage cells remained viable during the following cell cycle. Indeed, stable tetraploid cells can be generated from some cancer cell lines by treating them transiently with spindle poisons^41–43^. It is likely that mitotic slippage may not be an effective strategy in killing cancer cells that have mechanisms that can cluster or inactivate supernumerary centrosomes.

Interestingly, TRIP13 deficiency also promoted cell death in the presence of concentrations of spindle poisons insufficient for causing mitotic slippage (Fig. 5, Fig. S7). Although cell division could be completed, mitosis was shortened (Fig. S8) and displayed a significant increase in chromosome missegregation (Fig. 7). Micronucleation was also frequently observed in the daughter cells (Fig. 7). Micronuclei can be spontaneously generated in HeLa cells through multiple mechanisms, including lagging chromosomes during anaphase (the majority of cases), displaced chromosomes in metaphase and chromosomal bridges^44^. In the case of partial spindle disruption and weakened SAC, the origin of the micronucleation appears to be mainly from displaced chromosomes in metaphase (Fig. 7). Other studies indicated that chromosome missegregation caused by mitotic defects including reduction of MPS1 or BUBR1^45^, CENP-E^46^ or Spindly^47^ also induce cytotoxicity.

It is unclear why similar to mitotic slippage, most of the daughter cells survived the first cell cycle after partial chromosome missegregation in SAC-deficient cells. Cell death only progressively increased in the subsequent cell cycles (Fig. S8). It is possible that cancer cells such as HeLa can tolerate missegregation of both the entire genome and a small number of chromosomes for one cell cycle. Why cells with missegregation of a small number of chromosomes started to die in subsequent cell cycles is more difficult to explain. One possibility is that the presence of micronuclei can be source of further genome instability such as through chromothripsis.

An implication is that the different effectiveness of SAC in cancer cells may affect the outcome of spindle poison therapies. This is an important issue because in addition to spindle poisons, other emerging anticancer chemicals targeting mitotic regulators such as Aurora kinases, polo-like kinases or kinesin Eg5 (KIF11) also act by triggering protracted mitotic arrest. Consistent with the results that SAC only needed to be turned off transiently for synergism with spindle poisons (Fig. 6b), inhibiting MPS1 transiently with the inhibitor AZ3146 sensitized cells to spindle poisons (Fig. 8). Cell death appears to be caused by a similar mechanism as in SAC-deficient cells, characterized by chromosome missegregation and micronucleation (Fig. 8). These results are consistent with studies using other MPS1 inhibitors and spindle poisons^48–50^. It is possible that in addition to the components described here, other components of the SAC can also be clinically relevant drug targets.

## Supplementary information

Supplementary legdends

Figure S1

Figure S2

Figure S3

Figure S4

Figure S5

Figure S6

Figure S7

Figure S8

Video 1

Video 2

Video 3
