# Supplementary material for: Imbalance of the spindle-assembly checkpoint promotes spindle poison-mediated cytotoxicity with distinct kinetics
Source: Cell Death Dis. 2019 Apr 5;10(4):314. doi: 10.1038/s41419-019-1539-8 (PMC6450912; doi:10.1038/s41419-019-1539-8)
Supplement: Supplementary file 1 — Supplementary legdends [file 41419_2019_1539_MOESM1_ESM.docx]

**SUPPLEMENTAL FIGURE LEGENDS**

**Figure S1. Knockout of p31^comet^ increases sensitivity to spindle poisons.**

HCT116 (WT) and p31^KO^ cells were plated for clonogenic survival assay and incubated with NOC (11 ng/ml). The number of colonies was normalized to control. Mean±SEM of three independent experiments. Representative images are shown on the right.

**Figure S2. Mitotic slippage induced with MPS1 inhibitor.**

Mitotic HeLa cells were isolated by incubating with NOC for 4 h followed by mechanical shake off. The cells were pretreated with or without the proteasome inhibitor MG132 for 1 h as indicated and then treated with the indicated concentrations of MPS1 inhibitor AZ3146 (MPS1i), either in the presence or absence of the proteasome inhibitor MG132 for 3 h. Lysates were prepared and the indicated proteins were detected with immunoblotting. Actin analysis was included to assess protein loading and transfer.

**Figure S3. SAC-deficient cells resist mitotic cell death through mitotic slippage.**

**(A)** TRIP13-deficiency promotes mitotic slippage and reduces mitotic cell death. HeLa (WT), TRIP13^KO^, and TRIP13^KO^ expressing FLAG-TRIP13 were treated with buffer or PTX (8 and 31 ng/ml) for 24 h. Lysates were prepared and the expression of the indicated proteins was analysed with immunoblotting. Uniform loading of lysates was confirmed by immunoblotting for actin.

**(B)** TRIP13-deficiency promotes re-replication in the presence of spindle poisons. WT, TRIP13^KO^, and TRIP13^KO^ expressing FLAG-TRIP13 were incubated with buffer or the indicated concentrations of PTX for 24 h. The cells were then fixed and analysed with flow cytometry. The positions of 2N, 4N, and 8N DNA contents are indicated.

**Figure S4. TRIP13-deficient cell lines.**

**(A)** Expression of TRIP13 in WT and TRIP13^KO^ cells. Lysates from WT and TRIP13^KO^ cells from HeLa (left) or HCT116 (right) were analysed with immunoblotting. Uniform loading of lysates was confirmed by immunoblotting for actin.

**(B)** Conditional depletion of TRIP13. TRIP13^KO^ cells expressing AID-TRIP13 were incubated with either buffer or Dox and IAA together for 36 h. Lysates were prepared and analysed with immunoblotting.

**Figure S5. SAC-deficient HCT116 cells resist mitotic cell death through mitotic slippage.**

**(A)** WT and TRIP13^KO^ HCT116 cells were exposed to buffer or the indicated concentrations of NOC for 24 h. The cells were then fixed and analysed with flow cytometry. The positions of 2N, 4N, and 8N DNA contents are indicated.

**(B)** WT and TRIP13^KO^ HCT116 cells were exposed to buffer or the indicated concentrations of PTX for 24 h. The cells were then fixed and analysed with flow cytometry.

**Figure S6. Cell death starts to occur after one cell cycle following mitotic slippage.**

**(A)** Live-cell imaging of SAC-deficient cells. HeLa (WT), TRIP13^KO^, and TRIP13^KO^ expressing FLAG-TRIP13 (all expressing histone H2B-GFP) were exposed to buffer, NOC, or PTX. Individual cells were tracked using live-cell imaging. Key: interphase = grey; normal mitosis = black; mitotic slippage = red; truncated bars = cell death.

**(B)** TRIP13-deficient cells undergo rapid mitotic exit. Cells were exposed to PTX and analysed with live-cell imaging as described in panel A. The duration of mitosis in individual cells was determined (from DNA condensation to mitotic slippage or cell death).

**(C)** Mitotic slippage delays PTX-mediated cell death. Cells were exposed to PTX and analysed using live-cell imaging as described in panel A to quantify cumulative cell death.

**Figure S7. SAC-deficient cells are sensitive to a low concentration of spindle poison.**

**(A)** Depletion of TRIP13 in HCT116 promotes sensitivity to NOC. WT and TRIP13^KO^ HCT116 cells were exposed to buffer or NOC (11 ng/ml) for 24 h before gently washed. Colonies were fixed and stained after 14 days. The number of colonies was quantified (mean±SEM of three independent experiments). Representative images are shown on the left.

**(B)** SAC-deficient cells are sensitive to a sub-lethal dose of PTX. Equal number of HeLa (WT), TRIP13^KO^, and TRIP13^KO^ expressing FLAG-TRIP13 were treated with either buffer or PTX (2 ng/ml) for 24 h. After gentle washing, the cells were grown for another 14 days. Colonies were fixed, stained (representative images are shown on the left), and quantified (right). Mean±SEM of three independent experiments. Representative images are shown on the left.

**Figure S8. Aberrant mitosis induced by defective SAC and partial spindle perturbation promotes cell death only after the following cell cycle.**

HeLa (WT) and TRIP13^KO^ expressing histone H2B-GFP were exposed to NOC (11 ng/ml) for 24 h and analysed with live-cell imaging. The cells were washed and subjected to another period of live-cell imaging (24-72 h after initial NOC addition). Another batch of cells treated with NOC followed by washing was imaged from 72-120 h after initial NOC addition. Control cells without exposing to NOC were also imaged for 48 h and shown on the left. Key: interphase = grey; mitosis (from DNA condensation to anaphase or cell death) = black; multipolar mitosis or mitosis with segregation errors = green; truncated bars = cell death.

**SUPPLEMENTAL VIDEO FILES**

**Video 1.** **Mitotic slippage in TRIP13^KO^ cells after spindle perturbation.**

TRIP13^KO^ expressing histone H2B-GFP were exposed to NOC (100 ng/ml ) and analysed with live-cell imaging. Time scale in h:min.

**Video 2. Partial spindle perturbation promotes chromosome missegregation in TRIP13^KO^ cells.**

TRIP13^KO^ expressing histone H2B-GFP were exposed to NOC (11 ng/ml ) and analysed with live-cell imaging. Chromosome segregation errors can be observed during mitosis; and micronuclei can be observed in the daughter cells. Time scale in h:min.

**Video 3. Chemical inhibition of SAC promotes chromosomal missegregation after partial spindle perturbation.**

HeLa cells expressing histone H2B-GFP cells were exposed to MPS1i (0.5 µM) and NOC (11 ng/ml) and analysed with live-cell imaging. Chromosome segregation errors can be observed during mitosis; and micronuclei can be observed in the daughter cells. Time scale in h:min.
