# Supplementary figures and images for: Imbalance of the spindle-assembly checkpoint promotes spindle poison-mediated cytotoxicity with distinct kinetics
Source: Cell Death Dis. 2019 Apr 5;10(4):314. doi: 10.1038/s41419-019-1539-8 (PMC6450912; doi:10.1038/s41419-019-1539-8)

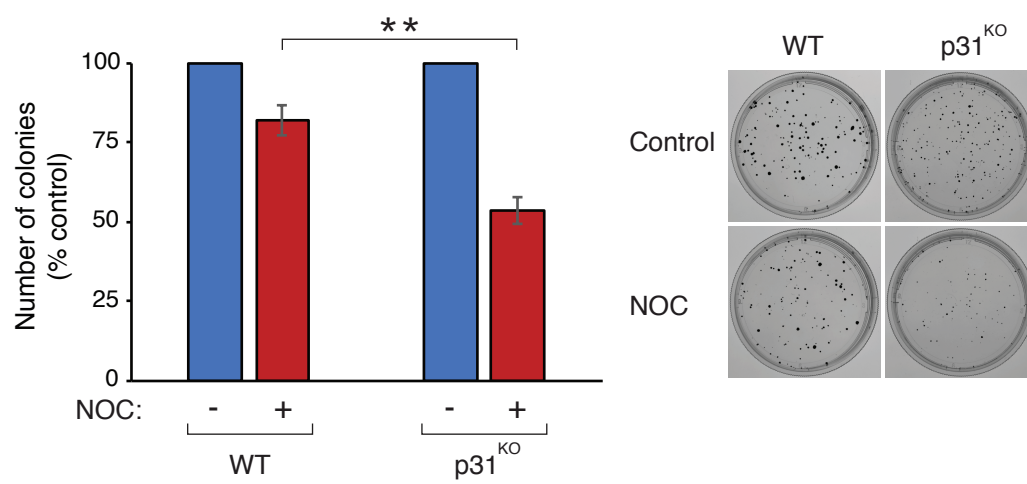

Supplemental Figure S1

Supplement: Supplementary file 2 — Figure S1 [file 41419_2019_1539_MOESM2_ESM.pdf]

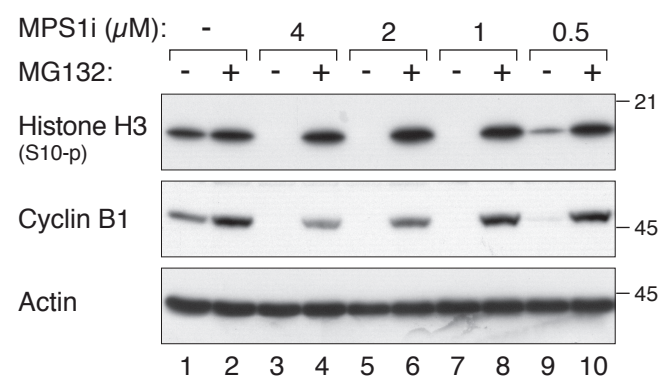

Supplemental Figure S2

Supplement: Supplementary file 3 — Figure S2 [file 41419_2019_1539_MOESM3_ESM.pdf]

# A

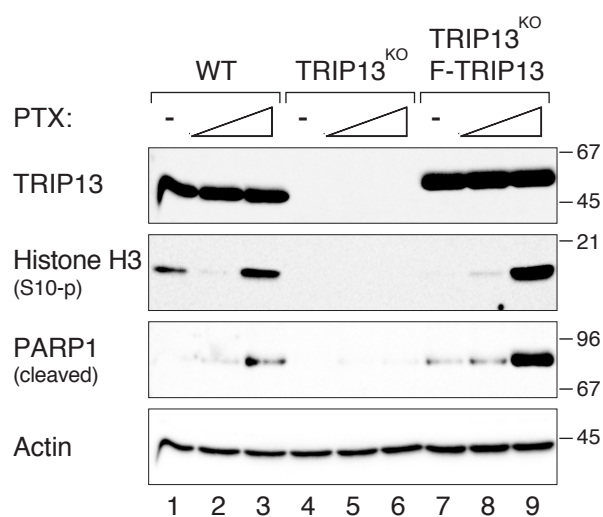

# B

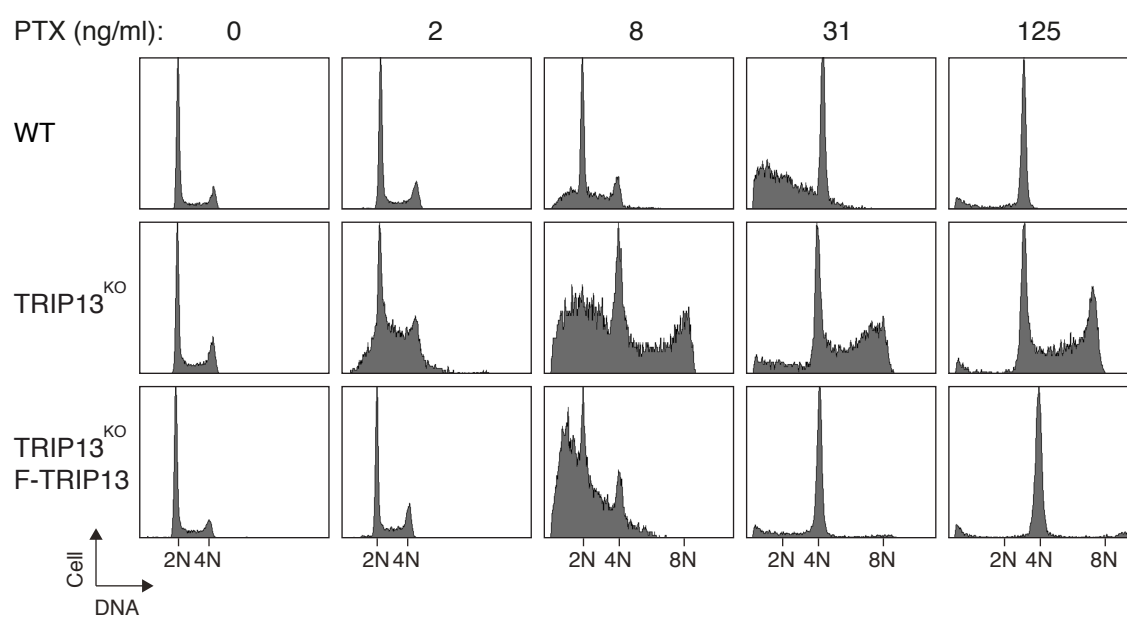

Supplement: Supplementary file 4 — Figure S3 [file 41419_2019_1539_MOESM4_ESM.pdf]

**A**

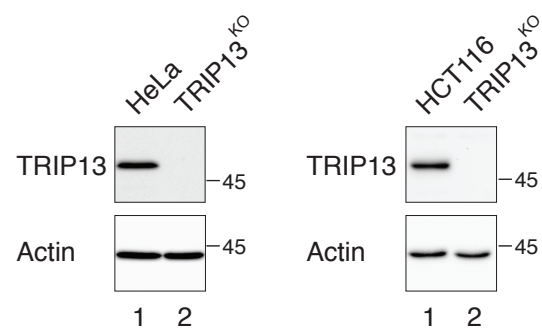

**B**

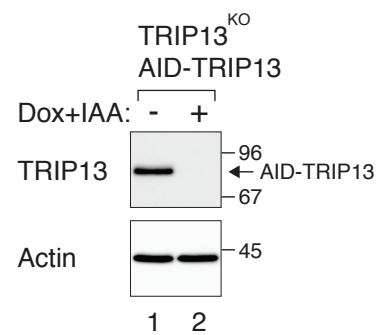

Supplement: Supplementary file 5 — Figure S4 [file 41419_2019_1539_MOESM5_ESM.pdf]

**A**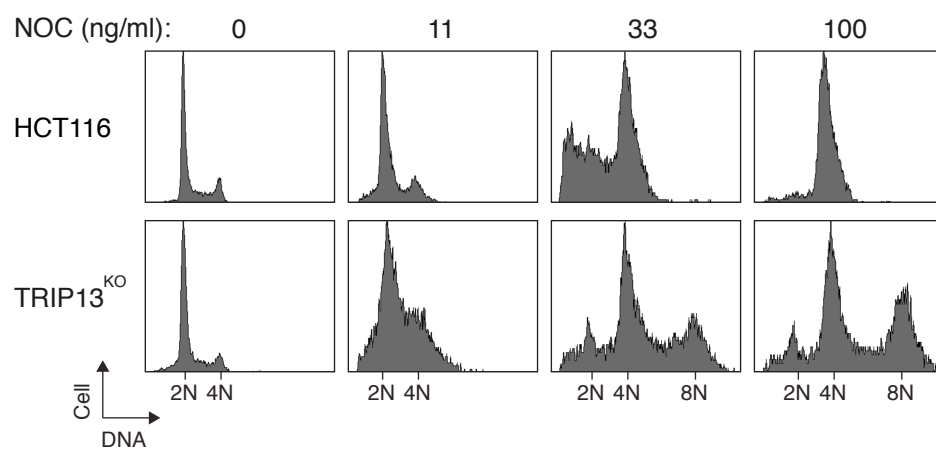**B**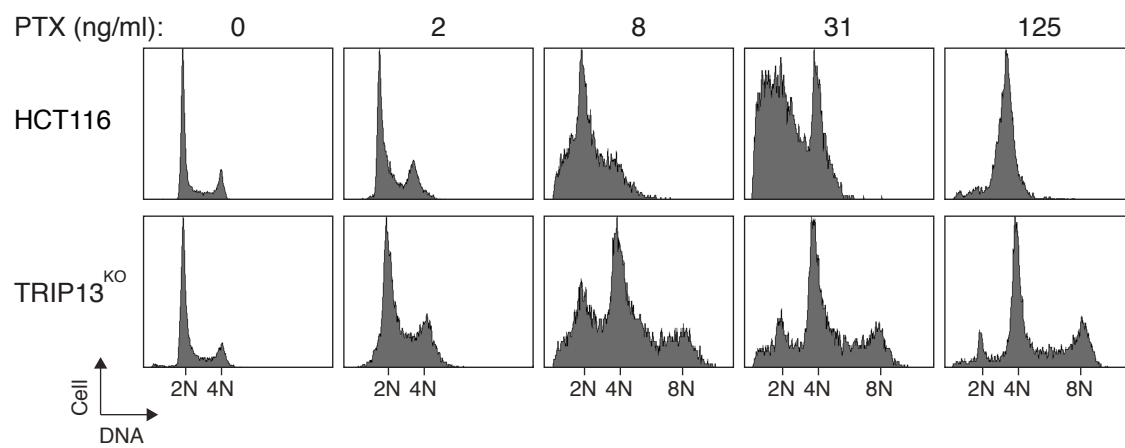

Supplement: Supplementary file 6 — Figure S5 [file 41419_2019_1539_MOESM6_ESM.pdf]

**A**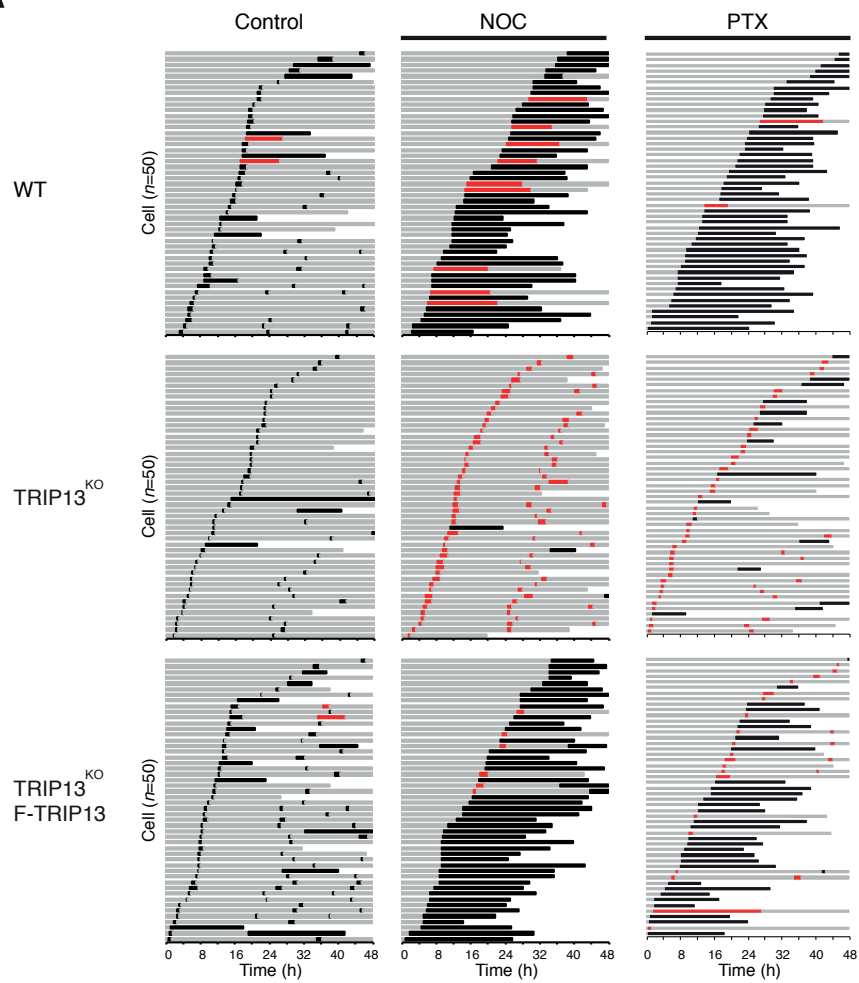**B**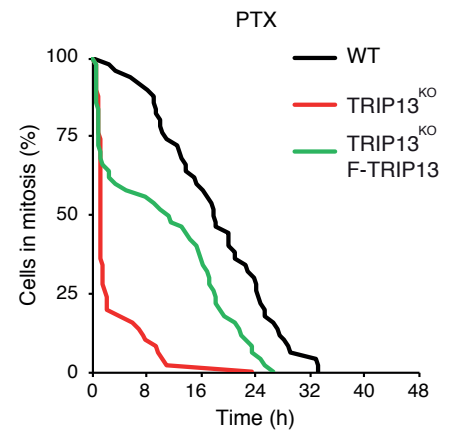**C**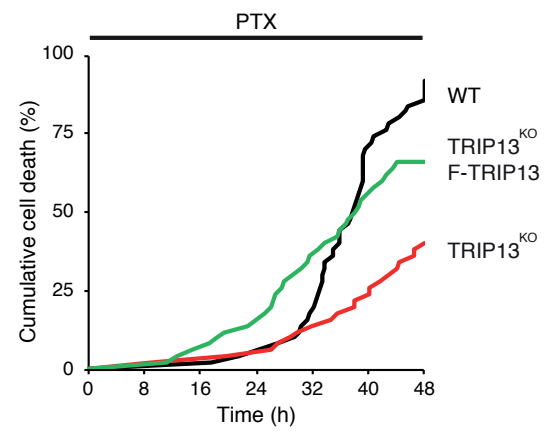

Supplement: Supplementary file 7 — Figure S6 [file 41419_2019_1539_MOESM7_ESM.pdf]

**A**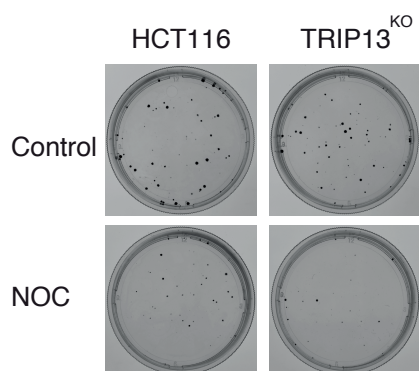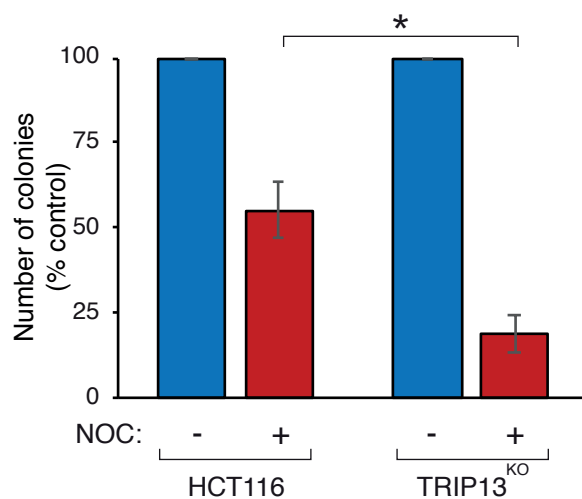**B**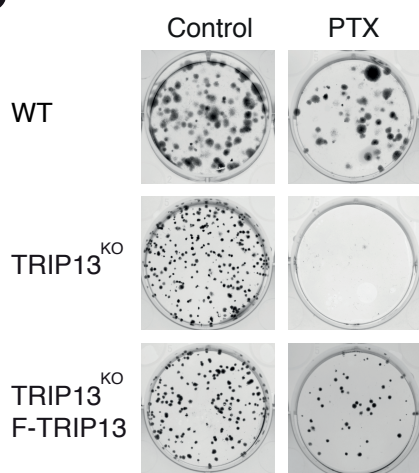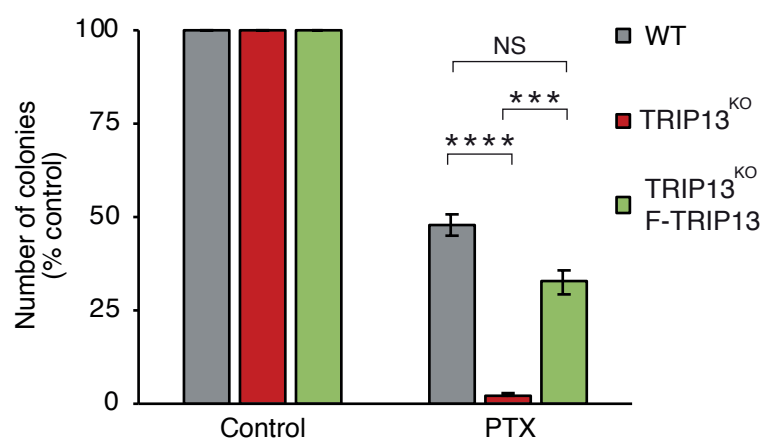

Supplement: Supplementary file 8 — Figure S7 [file 41419_2019_1539_MOESM8_ESM.pdf]

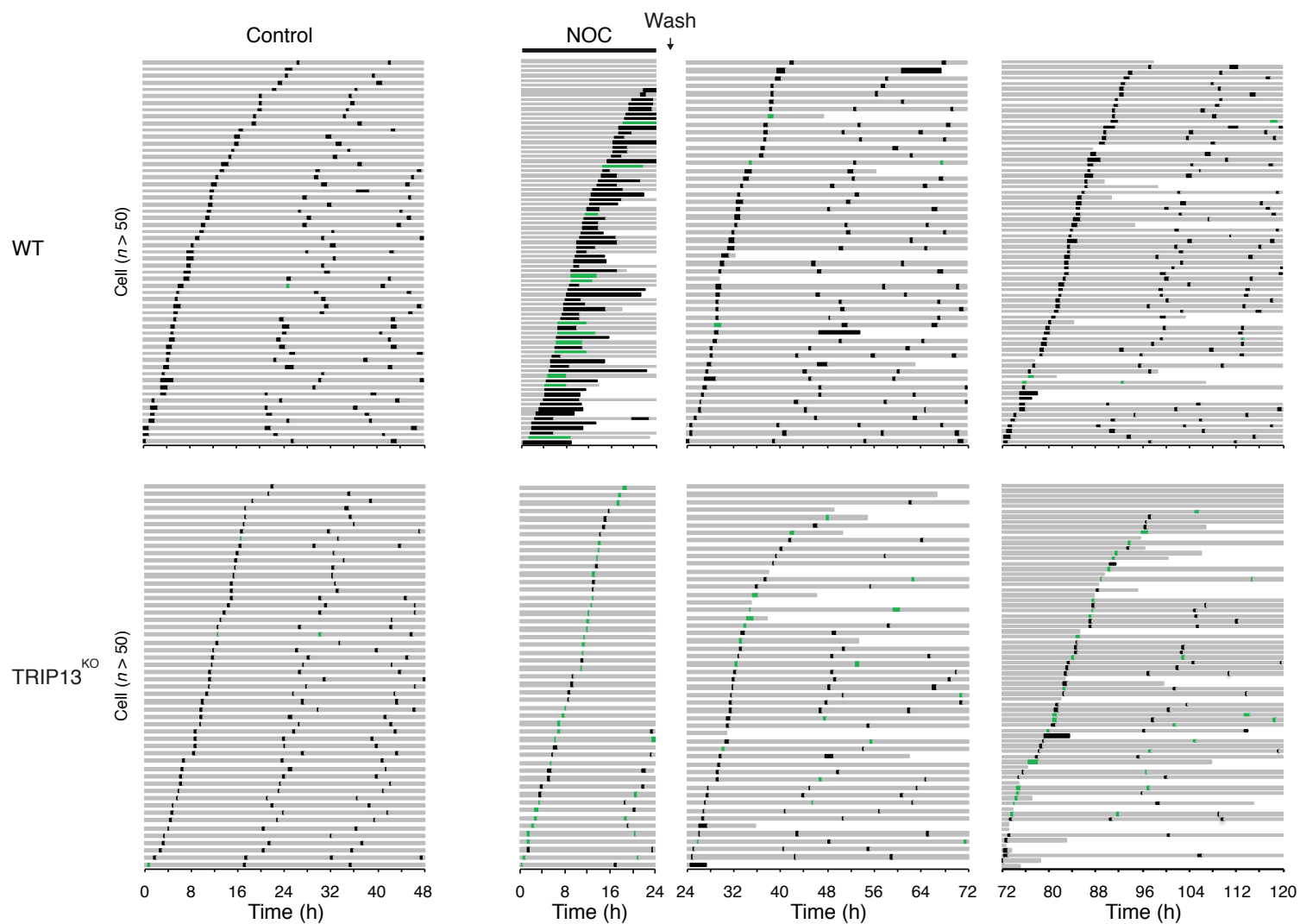

Supplemental Figure S8

Supplement: Supplementary file 9 — Figure S8 [file 41419_2019_1539_MOESM9_ESM.pdf]
